# Supplementary material for: Are carbon emissions trading and green financial instruments synergistic? -Comprehensive quantitative research based on content analysis
Source: PLoS One. 2024 Mar 7;19(3):e0298601. doi: 10.1371/journal.pone.0298601 (PMC10919601; doi:10.1371/journal.pone.0298601)
Supplement: S1 Data — (ZIP) [file pone.0298601.s001.zip › Data/地级市Z_F工作报告.zip!/║┌┴·╜¡/┤≤╟∞╩╨/└┤╫╘╣1⁄2╓┌║┼í╛┬φ┐╦╩2╛▌═°í┐_2005.docx]

各位代表：

　　现在，我代表大庆市人民政府，向大会作政府工作报告，请予审议，并请市政协各位委员提出意见。

　　一、2004年工作回顾

　　过去的一年，我们在市委领导下，认真贯彻党的十六大和十六届三中、四中全会精神，紧紧围绕建设高科技现代化城市和全面建设小康社会目标，坚持解放思想，求真务实，圆满完成市七届人大三次会议确定的20项大事实事年度目标和6个方面重点任务。

　　---经济实现快速增长。

　　全市地区生产总值实现1239.5亿元，同比增长10.2%。地方财政总收入实现67.7亿元（含杜尔伯特自治县），同比增长58%。固定资产投资238.2亿元，同比增长10.6%。农业和农村经济健康发展，实现农业增加值40亿元，同比增长14%；粮食总产34.4亿斤，同比增长34.7%；畜牧业实现增加值20.4亿元，占农业增加值的51%；培训农民工3.9万人，转移劳动力9万人，实现劳务收入4.5亿元。工业经济持续快速增长，实现增加值1008亿元、利税943.4亿元，同比分别增长9.8%和23.2%。油田公司生产原油4640万吨，实现增加值802亿元，同比增长3.9%；中省直石化大企业120万吨加氢裂化、60万吨乙烯、20万吨高压聚乙烯等项目建成投产，实现增加值70.3亿元，同比增长34.4%；石油管理局推进集团化、专业化经营，市场竞争能力明显增强，实现工业增加值27.2亿元，同比增长25.4%。以高新区为龙头的地方工业增势强劲，新开工建设项目239个，投产158个，地方工业实现增加值106亿元，同比增长40.5%。第三产业稳步发展，实现增加值151.1亿元，同比增长14.2%。连锁超市等新型业态快速发展，社会消费品零售总额实现130亿元，同比增长20.4%。民营经济发展步伐加快，实现增加值155亿元、利税23亿元，同比分别增长15%和25%。

　　---改革开放富有成效。

　　石油石化大企业分离办社会职能工作取得突破，125所企办中小学、9个教育机构和4个公安机构移交地方。免征农业税试点涉及的乡镇机构、乡村财政管理体制、农村义务教育管理体制“三项改革”基本完成，精简乡镇行政事业机构218个、减少编制1031个、分流人员761人。全面落实粮食补贴政策，兑付农民补贴资金9729万元，兑付率99.4%。国有企业下岗职工基本生活保障向失业保险并轨扎实推进，已有1.2万人享受经济补偿金。粮食企业改革全面展开，国有资产置换和国企职工身份置换稳步推进。完善集中收付、投资评审和政府采购制度，节约财政支出3.7亿元，增收7.8亿元。农村信用社改革顺利进行，已清理不良贷款本息合计2.8亿元，增资扩股5.3亿元。对外开放成果丰硕，招商引资共引进项目541个，到位资金57亿元。在美国、俄罗斯、日本等9个国家设立了经贸联络处。扶持企业开拓境外市场，拥有对外进出口权的企业发展到280家，出口产品增加到132个品种，出口国家和地区达到73个，进出口总额实现1.3亿美元，外资实际到位2411万美元，同比分别增长67.9%和31%。

　　----地企合作更加紧密。

　　联合开发小油田，有力拉动了县域经济增长。依托大企业系统工程，完善宏伟、兴化园区载体功能，共同引进开发了一批工业项目。大企业投资16.2亿元，实施路桥、场馆、污水处理、老区改造等城市建设项目11个，治理主城区湖泊2个、植树造林1.4万亩，在完善城市功能、改变城市面貌等方面做出了重要贡献。共同妥善处理有偿解除劳动合同人员、退养家属等群体上访问题，联手整治油田治安秩序。共同向上争取国家资源型城市经济转型试点。地企联合更加广泛、深入。

　　----城乡建设步伐加快。

　　投资82亿元新建续建城市基础设施项目359个，施工面积405.5万平方米。哈工大工业研究院、铁人纪念馆等52项市级重点工程完成当年建设任务。通县乡村公路建设加快，完成硬化路面1002公里。拆除违法违章、占压油气管线建筑23万平方米，完成45个老居住区348栋住宅楼改造，建设流动人口居住区5.4万平方米，我市改善居民居住条件项目荣获“中国人居环境范例奖”。生态市建设取得明显成效，实施龙凤湿地自然保护区、三永湖、果午泡、乘风湖、东风泡、新潮湖综合治理工程，启动建设生态园12个，主城区退耕还林1万亩，新建城市绿地797万平方米，植树造林24.8万亩，油田植被恢复20万亩，草原禁牧400万亩。经营城市效果显著，土地经营收益4亿元，其中招拍挂竞价出让土地95宗92.9公顷，收取出让金2.9亿元，比基准地价提高了40%。

　　----社会事业不断进步。

　　科技创新能力进一步增强，完成科技攻关项目522个，研发自主知识产权项目513个，推广应用科技成果514项，高新技术企业发展到280户，高新技术产业产值占地方工业产值比重达到26%。教育整体水平进一步提高，哈医大大庆校区落成招生，大庆师范学院正式挂牌，城市拥有高校数量跃居全省第2位，在校生达5.3万人；17中、28中通过省级示范高中评审，市区基本普及高中教育，高考升学人数首次突破万人，万人在校大学生达到204.2人；改造农村中小学危房68所，办学条件明显改善；大庆以全省总分第一的成绩通过省二类城市语言文字评估验收。医疗卫生事业健康发展，重点加强眼病、肝病、心脏病等特色医疗专科建设，第二医院成为北京佑安医院大庆分院，在传染病医院综合实力排位中名列全省第一，进入全国十强；实施新型农村合作医疗、社区卫生服务、结核病防治工程和乡村医生培训计划，疾病预防控制和公共卫生监督体系基本建立。文化体育事业蓬勃发展，有9部作品荣获国家级奖项，“大庆之冬”和“激情之夏”等群众性文化活动深入开展；运动员在国际和国内重大比赛中获得优异成绩，群众性体育运动广泛开展。创建国家文明城工作全面启动，积极开展“九个百工程”，精神文明建设向广度和深度推进。

　　　 ----人民生活水平提高。

　　城镇居民人均可支配收入达到12324元，同比增长11.7%。扣除“一免两补”政策性因素影响，农民人均纯收入达到2284.6元，同比增长15.4%。安置就业4万人，城镇登记失业率4.4%。解决拖欠农民工工资4635万元，占总额75%。参加养老保险、失业保险、医疗保险、工伤保险、生育保险人员同比增加5.6万人。城镇居民最低生活保障人均补差86.2元，比上年提高26.4元。城乡居民储蓄存款余额467亿元，人口自然增长率4.1‰，全市居民消费价格总指数为103.5%，城市人均住房使用面积达到23.3平方米，汽车、通信、旅游等新型消费持续增长，城市恩格尔系数27.8%，人民生活向更加富裕阶段迈进。

　　大力加强社会治安综合治理，严厉打击各种违法犯罪，强化重点行业安全管理，妥善解决群众信访问题，防控打击“法轮功”等邪教组织，社会稳定，政治安定，人民群众安居乐业。此外，新闻出版、民族宗教、文史档案、人防、国防后备力量建设等项工作，都取得了新成绩。

　　各位代表，过去的一年，是我市经济发展和社会进步较快的一年。这是市委正确领导的结果，是几大班子及全市人民团结奋斗的结果，是石油石化大企业紧密协作的结果，是全市上下真抓实干的结果。在此，我代表市政府，向全市各族人民，向人大代表、政协委员，向各民主党派、人民团体、各界人士，向中省直驻庆各单位、人民解放军和武警部队，以及所有关心支持大庆发展的同志们，表示崇高的敬意和衷心的感谢！

　　各位代表，过去的一年，虽然取得了显著成绩，但是我们必须清醒地看到，全市经济社会发展和政府工作中还存在不少困难和问题。主要是：支撑可持续发展的产业基础不够牢固，体制、机制和科技创新的任务仍很艰巨；发展环境需要进一步优化，城市功能有待进一步完善；县域经济发展水平不够平衡，城乡居民收入差距较大；资源保护欠账较多，生态建设与环境治理任务繁重；经济外向度较低，招商引资质量有待提高；城市商务运营成本较高，公共资源调控与科学配置需要进一步加强；政府职能转换存在差距，服务意识和依法行政能力有待增强；就业和再就业压力较大，城乡部分低收入群众生活困难，社会保障体系需要进一步完善，社会稳定任务还十分艰巨。这些问题，我们一定高度重视，立足当前，着眼长远，统筹考虑，逐步解决。

　　二、2005年主要任务

　　新的一年，是全面实现“十五”计划目标的重要一年，是构建大庆全面发展战略新高地的起步之年。今年市政府工作总体要求是：以邓小平理论和“三个代表”重要思想为指导，认真贯彻市委六届三次全会精神，坚持以人为本，坚持求真务实，坚持科学发展观，以构建大庆全面发展战略新高地为目标，以发展经济改善民生为主题，以项目建设为中心，以优化环境为保障，切实推进事关大庆当前和长远发展的经济和社会事业。全市经济和社会发展主要预期目标是：大庆市地区生产总值确保增长9%，力争10%。全社会固定资产投资增长15%左右，社会消费品零售总额增长11.5%以上，居民消费价格总指数上涨幅度控制在4%以内，地方财政总收入按可比口径比上年预算增长12.4%，外贸进出口总额增长20%，城镇居民人均可支配收入增长8.7%，农民人均纯收入增长5%，城镇登记失业率控制在4.8%以内，人口自然增长率控制在5.3‰以下。

　　实现上述预期目标，要科学统筹、清醒把握政府工作的主攻方向。

　　第一，必须不断丰富和深化大庆经济发展内涵，打造接续产业新优势。既要保持较快经济增长速度，更要提高经济增长质量，由单一资源型经济向复合型经济转变。努力构造石油、石化、管理局、高新区和各县（区）组成的五极经济板块，在确保石油产业这一全国战略高地的同时，努力把石油化工、天然气化工、乳制品加工、粮食及畜产品加工、电力工业、机械制造、电子信息、新材料、纺织业等建成几十亿、上百亿、超千亿的支柱产业。争取国家在大庆实施资源型城市经济转型试点，建立资源开发补偿机制和衰退产业援助机制；优化投资、产业、消费结构，大力发展接续产业；挖掘人才、教育、科技、文化、卫生、体育等方面资源，努力把资源优势转化为产业优势。

　　第二，必须坚持市场经济取向，全面优化城市发展环境。城市环境是一个地方的生产力，是推动经济社会发展的内在动力。政府要转变职能，推进体制创新、组织创新和制度创新，积极履行经济调节、市场监管、社会管理和公共服务职能。重视发展科技教育事业，保护吸引和培养专业人才，打造城市核心竞争力；调整不合理的利益格局，增强市场在资源配置中的基础性作用，使大庆特有的经济实力、城市品牌和中省直大企业优势得到充分发挥。不断改善城市硬件环境和软件环境，增强城市发展活力和综合实力。

　　第三，必须统筹城市与农村、经济与社会协调发展，努力构建和谐社会。离开四县农民的小康就没有全市的小康，离开郊区的现代化就没有大庆的现代化。要努力缩小城乡差距，合理引导要素向县（区）倾斜，形成协调的产业布局，加快工业化、城市化、现代化的进程。要重视西城与东城协调发展，努力实现经济、社会、生态三个效益的统一。

　　第四，必须坚持建设城市与管理城市并重，提升城市现代化水平。管理城市是政府的重要职责。坚持以规划为龙头，抓住城市空间布局实施“东移北扩”战略调整机遇，综合考虑城市功能布局，拓展城市发展空间。推进城市管理体制创新，强化规划、交通和环卫管理，不断提高城市管理和公共服务水平。

　　第五，必须全力维护好人民的根本利益，使广大群众在改革与发展中得到更多实惠。把群众的呼声作为指导工作的第一信号，把关心和服务群众作为第一职责，把群众的评价作为衡量工作政绩的第一尺度，在土地征用中依法维护好农民利益，在城市改造建设中维护好居民利益，在全面提高人民生活水平中照顾好困难群体利益，认真解决涉及群众切身利益的突出问题。为此，要着力做好以下5方面工作。

　　（一）紧紧扭住项目建设，加快城乡经济发展

　　壮大工业经济。抓住我省建设哈大齐工业走廊的契机，推进在建项目，抓好新上项目，论证近期项目，规划远期项目，引进大高外项目，促进工业壮大规模、提档升级，确保实现工业增加值1065亿元，力争突破1100亿元，增长8.3%。积极配合油田公司实施科技兴企、人才强企和低成本三大战略，努力创建“百年油田”，为非油产业发展提供支撑。支持中省直大企业加快80万吨乙烯、30万吨聚丙烯、20万吨醋酸、10万吨ABS、8万吨顺丁橡胶、10万吨干气制乙苯、56万吨合成氨和76万吨尿素等14个总投资超百亿元大项目的建设，确保实现增加值134亿元，同比增长25%以上。围绕解决石化原料不足和品种不全的问题，研究论证对现有的原油加工工艺路线进行改造，开发乙烯、丙烯、丁二烯和纯苯、邻二甲苯、对二甲苯，增加基础有机化工原料，延长石化产业链，为扩大石化产业规模奠定基础。支持油田企业加快天然气开发，与大企业共同做好深加工利用规划，研究论证165万吨天然气—甲醇—烯烃—乙二醇联合装置、60万吨芳构化装置和150万吨二甲醚装置等天然气化工大项目，努力把大庆建设成为天然气化工生产基地。

　　加快推进地方工业“百项工程”，新建续建工业项目150个以上，力争达到200个，实现地方工业新跨越。发挥高新区的龙头作用，按照高新技术产业主导区、对外开放先行区、现代化城市示范区的发展定位，提升科技创新能力、支柱产业竞争力、高新技术产业带动力，提高对全市经济增长的贡献率。重点推进大豆产业园，支持中国航天科技集团大庆航天科技园启动建设。发挥县（区）优势，壮大特色园区。加快建设林源轻纺工业园，以及注塑、皮革、橡胶、精细化工等在建专业园区，打造非油工业产业集群。提升杜蒙德力戈尔、红岗铁人生态、林甸永青、肇源港桥、让胡路民营科技、肇州丰乐等综合性工业园区功能，启动建设玉米深加工、医药等新园区，构筑地方工业发展新格局。加大对现有企业的服务力度，选择20户发展潜力大、成长性好的企业进行重点扶持，打造地方工业强企。确保实现地方工业增加值136亿元以上，同比增长40%。

　　做强农业经济。加快发展以产业化为核心的工业主导型农村经济，努力实现农业增效、农民增收、农产品竞争力增强。大力推进以农转牧为重点的主辅换位，健全资金支持、科技推广、繁育改良、防疫灭病、饲料供应、风险防范等畜牧业社会化服务体系，推进标准化、规模化和集约化养殖。力争实现畜牧业增加值25亿元，占农业增加值的54.3%。加快农业产业化进程。坚持强龙头，壮基地，协调好产加销之间利益关系，发展壮大乳业、生猪、大鹅、玉米等10个产业链，使农产品在精深加工中增值，进而促进农民增收。建设抗旱保收田。总体规划、分步实施、先易后难、区域推进。投资支持新打机电井1010眼，上喷灌设施2400台套，争取用4年左右时间，在原有150万亩基础上建成400万亩保人畜用粮、保龙头企业原料、保城市“菜篮子”的抗旱保收基本农田。实施良种化工程，推行标准化生产，扩大绿色特色农产品基地面积，发展优质高产高效农业。推进劳动力转移，落实好中央、省关于转移农村富余劳动力的各项政策，加大财政支持力度，探索建立农民工培训转移的长效机制，提高劳动力转移的组织化、技能化、合同化水平，发展劳务经济。建立转移平台和基地30个，培训农民工4.5万人，转移劳动力7.5万人，创收10亿元。加快培育发展农民专业合作经济组织，健全社会化服务体系，畅通农产品流通渠道。

　　繁荣第三产业。抓好第三产业发展规划编制和论证，调整优化布局，提高发展水平。积极培育市场，主要是建设大庆石化产品批发市场、太阳升粮食批发市场、让区汽配批发市场、萨中建材批发市场、杜蒙林甸乳品批发市场、铁西旧物批发市场。努力发展物流，以建设覆盖黑龙江省西部、辐射东北地区、连接东北亚的流通大市场为目标，积极论证规划，争取与油田公司和石油管理局联手建设大庆石油物资物流园，与石化公司和石化总厂联手建设大庆石化物流园，依托肇源港和松花江大桥建设港桥物流园。开发旅游产业，主要是利用地热资源，做大绿色温泉游品牌；以水为重点，做活湿地风情游品牌；以油为主题，做深石油文化游品牌。精心保护开发连环湖等生态旅游资源，建成独具特色的旅游热线。搞好创业追踪园、油田科技博物馆、石油会战传统教育基地建设，启动“北国之春”绿色温泉城项目。搞活商贸流通，启动东风中学原址商业中心区建设，完善已初具规模的15条特色商业街，逐步把经六街建成集购物、娱乐、餐饮、旅游于一体的特色商贸一条街。发展便民连锁等新型业态，以及金融、保险、信息等现代服务业，规范发展律师、会计、咨询、策划、资产评估、产权交易、房屋租赁等中介组织。

　　（二）切实重视民生问题，提高人民生活水平

　　做好就业和再就业工作。落实财政投入、小额贷款、免费培训、税收减免等各项促进就业政策，强化就业服务，改善就业环境。通过发展服务业、建筑业、劳动密集型产业、劳务输出和置换购买等途径开发就业岗位，新增城镇就业3.5万人。抓好下岗失业人员再就业技能培训，推广职业资格认证制度，鼓励企业优先录用本地下岗人员，培训下岗失业人员1.2万人，确保培训合格率达到90%以上，再就业率达到60%。加强劳动力市场管理，依法维护劳动者合法权益。关注新失业群体，高度重视高校毕业生、复转军人的就业指导和服务。

　　进一步完善社会保障体系。全面推进养老保险、失业保险、医疗保险、工伤保险、生育保险。突出抓好国有企业下岗职工基本生活保障向失业保险并轨工作，确保完成并轨目标，维护职工利益，保持社会稳定。积极扩大私营企业、个体工商户和灵活就业人员参保覆盖面。逐步建立农村社会保障体系和低保制度。认真落实“七位一体”救助办法，完善低保救助政策，适当提高低保标准，逐步提高低保对象的人均补差值。调整廉租房救助办法，由实物配租向货币化补贴转变。建立低保大病救助基金，对患大病的低保对象进行医疗救助。强化公共财政职能，努力扩大城镇中等收入群体比重，年内适当提高机关事业单位工作人员绩效工资水平。

　　切实维护农民利益。认真落实“一免两补”政策，加强农资市场和价格管理，防止农民负担反弹，促进农民增收。梳理财政分配关系，认真落实国家和省扶贫开发政策，做好农村贫困户、五保户、受灾户的扶贫救济工作。搞好农村敬老院的规划和改造，用3年时间全面完成15处农村敬老院的改造任务。继续抓好贫困村脱贫，争取51个年人均收入低于1300元的贫困村全部实现脱贫目标，年人均收入达到1600元以上。实施农村人畜饮水解困工程118处，解决6万人饮水困难。

　　全面加强社区建设。完善社区基础设施，重点抓好老年文体活动场所建设。推进城市管理和服务重心下移，增强社区自治、保障、管理、服务、稳定、教育等功能，为居民提供方便快捷的就业、救助、文体、金融、医疗、邮政、信息、配送、司法服务。加强社区组织建设，强化社区自我管理能力。

　　提高城乡卫生医疗水平。加强以疾病预防控制体系、疫情监测报告网络体系、突发公共卫生事件应急救助体系为重点的城乡公共卫生体系建设。加快市级医院资源整合，创建和完善设备先进、技术领先、各具特色的治疗中心。推进爱心医院和社区卫生服务工作。加大对农村医疗卫生事业的投入，改善乡镇卫生院、村卫生所的基础设施和基本医疗条件；通过定向培养、卫生下乡等多项措施，提高农村医疗卫生队伍素质；逐步扩大农村新型合作医疗覆盖面，探索贫困人员医疗救助办法。

　　推进城中村和棚户区改造。积极稳妥抓好红旗村、澳龙小区、中原路北平房区、勤奋地区棚户区动迁改造，建设会战东街二期流动人口居住区，新建经济适用住房和廉价廉租房32.2万平方米，解决1万名流动人口和1552户低收入家庭居住问题。

　　落实信访工作领导责任制。完善市领导信访接待日制度，政府班子成员每周集中接待一次来访群众，及时调处各种矛盾纠纷。发挥市长热线电话受理中心作用，妥善处理群众来电反映的问题。落实分级负责的信访工作责任制，高度负责地对待群众信访问题，尽量把问题解决在基层，矛盾化解在当地。立足解决群众反映的实际问题，建立长效机制，坚持依法治访，重点解决好城市拆迁、企业改制、土地纠纷、农民工工资等热点问题，严格控制集体访和越级访，预防和妥善处置群体性事件及突出信访问题。

　　（三）营造良好环境，增强城市竞争能力

　　全力优化服务环境。开展“诚信政府”、“诚信企业”、“诚信大庆人”系列活动，从一点一滴细节做起，增强大庆对投资者的亲和力、公信力，优化人文环境。认真落实招商引资、鼓励投资的各项政策，在土地、收费等方面，不断丰富完善有利于企业发展的招商引资政策，优化政策环境。发挥外来投资者投诉中心作用，完善新企业创办服务中心职能，真正落实“一条龙”服务、“一站式”办公，优化政务环境。切实发挥政府部门作用，加大市场监管力度，努力消除部门和行业垄断等地方保护主义行为，为投资者创造公平、公正的发展平台，优化市场竞争环境。清理行政审批事项，严肃处理和防止侵犯企业利益的行为，确保投资者人身财产安全，优化法治环境。

　　完善城市基础功能。重点抓好39个新建续建城市基础设施及功能项目。在道路交通方面，着眼构建高速、便利、快捷的立体化交通体系，加快301国道绕行线建设，完成萨大路、经九街延伸、肇肇路、让杜路等城乡公路工程。借助滨洲铁路提速改造，建成12座平改立跨线桥。在城区改造方面，实施西寨小区、中林小区、富强小区、庆新小区等20个老居住区环境改造，全面完成老居住区改造任务。启动滨洲新村、科学家村、北辰绿色家园二期住宅工程建设。新建住宅一律推行分户供热和天然气入户。开工建设大剧院、科技馆、青少年宫等文化设施。

　　加强城市经营管理。按照统一规划、连片开发、总量控制、政府垄断、集中管理、竞价出让的原则，经营好城市土地，实现城市升值。组建国土资源收购储备管理中心，建立“一个渠道进水、一个池子蓄水、一个龙头放水”的供地机制。公开招标、拍卖东干路、西一路等50条街路保洁权和重点地段小广告治理权，推行城市户外广告、出租车、长途客（货）运线路经营权和街路、广场、公交站亭冠名权有偿使用。开展“城市管理年”活动，实施城市亮化、净化、美化、绿化工程。消灭中宝路、中兴北街等9条无灯路，重点对经六街安全岛、301国道与世纪大道交叉三角区等2个节点5座桥9条商业街进行亮化。新建和完善阳光绿苑、奔腾转盘等34个生态园，启动东干线、西干线等4条城市绿廊带建设，完成主城区退耕还林5500亩，新建城市绿地520公顷。完善地名标志设施建设，搞好路灯、广告板、公厕、垃圾箱、花池等附属设施景观美化。加大环境卫生治理力度，全面提高城市管理水平。

　　加快生态恢复治理。抓好堵西北风口、嫩江沙地造林、农田草原防护林工程，植树造林16万亩。全面实行草原休禁牧，封区育草30万亩，草原改良20万亩。开工建设东城水库，启动地下水库建设，继续搞好果午泡、东风泡、新潮湖、龙凤湿地治理，净化水环境。抓好生产污水、生活垃圾、环境噪声等污染防控，推行清洁生产，确保西城区污水处理厂9月初竣工投入使用。发展循环经济，强化节能意识，制定能耗标准，加强政策引导，建设资源节约型社会。

　　（四）全面推进改革开放，大力抓好招商引资

　　深化规范企业和事业单位改革。支持石油石化未上市企业建立现代企业制度。通过国有资产投资公司运作,与大企业共同参股,组建企业,发展产业。进一步深化地方企业改革，推进粮食企业产权制度改革，解决好一些企业“股份后、出售后、兼并后、破产后”的遗留问题，促进企业健康发展。全面推进文化、教育、卫生、体育等事业单位改革。重点在市区中小学用人制度和分配制度改革上取得突破，对政府举办的普通中小学、职业中学、特殊教育学校，全面推行校长聘任制和教职工聘用制，实行岗位管理。同时创新分配机制和评价机制，真正做到人员能进能出、职务能上能下、待遇能高能低。

　　提高招商引资质量。以“项目建设年”、“招商引资年”为牵动，创新招商引资工作思路，力争在引进高科技项目、大项目和外向型项目上取得突破。围绕大庆比较优势和发展接续产业的主攻方向，开展重点招商、定向招商、专业招商、以商招商，推行市场化招商。以引进战略投资者为重点，争取引进1-2家国内和世界500强企业；突出高新技术产业，争取在石化、新材料、电子信息、装备制造、现代物流、旅游、现代农业等领域引进一批高水平项目；瞄准发展外向型经济，争取在吸引外资和外向型企业上实现新突破。充分发挥各级领导干部、各单位、各部门的潜力，发挥基层和企业的创造性，发挥驻外机构的作用，举全市之力招大商、引大资。组织好日本世博会大庆活动日、大庆—新加坡经济合作对接会、参加哈洽会等重点招商活动。借助石油石化大企业优势，利用存量资产，开展合作，引进项目。鼓励联谊、华科、日月星等上市企业与跨国公司优势互补，强强联合。完善招商引资考核和激励办法，推进招商引资扎实深入、健康有效地开展，力争引进亿元以上项目30个，引进市外资金比上年增长5.3%。

　　大力发展对外贸易。抓住全省对俄经贸合作战略升级启动年机遇，重点抓好俄罗斯克市大庆商品展示批发中心建设，推进中国装饰材料城建设，逐步把俄罗斯克市发展成为我市对俄商品批发转运基地。加快建设石油机械、纺织品、精细化工、食品四大出口基地，发展跨国流通企业，大力开拓俄罗斯市场，巩固中东、欧美市场，开发中亚新兴市场，拓宽对外贸易和国际合作渠道。扶持有基础、有潜力的外经贸企业做大做强，培育外向型骨干企业。积极申报建设出口加工区、内陆港和保税仓库，打造三位一体的出口加工企业发展平台，构筑外向型经济发展新优势。

　　推动民营经济提档升级。在市场准入、进出口、使用外汇、参与政府采购和招投标、高新技术企业认定、申报政府计划项目、科技奖励、取得许可证和资质等级证书及引进人才等方面，享有与国有企业同等待遇。支持有条件的民营企业上市融资，壮大实力；鼓励民营企业通过租赁、参股、兼并、收购等形式，参与国有企业改组、改造。组建中小企业协会和乳业、化工、畜产品加工、建筑建材等行业协会，支持成立外来投资者商会，维护民营企业权益。大力推进民营企业技术创新，培育名企、名牌、名品。启动诚信体系建设工程，从重点行业和重点企业起步，建立企业信用记录，坚决把信用差的企业清出大庆市场，形成有效的守信受益、失信惩戒机制。实行领导干部联系民营企业大户和专家包扶民营企业制度，努力为民营企业发展搞好服务。

　　（五）加强民主法制建设，繁荣各项社会事业

　　自觉接受人民监督。认真贯彻人大及其常委会决议，自觉接受监督。落实重大事项报告制度，接受审议。政府组成人员认真做好述职，虚心听取评议，及时加以整改。主动加强与政协及各民主党派、工商联和无党派人士的联系。认真负责地办理人大议案、建议以及政协提案，切实提高承办质量和水平。全面落实党和国家的民族宗教政策，做好侨务和对台工作。巩固和扩大基层民主，完善居民自治和村民自治，保障人民群众能够直接行使民主权力。认真贯彻《行政许可法》和《大庆市全面推进依法行政五年规划》，增强依法行政意识，规范行政许可行为，努力提高政府的法制化管理水平。发挥行政投诉受理中心作用，坚决查处公务人员行政违法行为，追究不作为、乱作为的责任。坚持科学民主决策，邀请人大和政协专门委员会、政府法律顾问、群众代表列席政府常务会议。建立和完善重大问题集体决策制度、专家咨询制度、社情民意反馈制度、社会公示和听证制度、决策责任追究制度。实行政府新闻发言人制度，及时对全市经济发展战略、重要发展规划、重大建设项目、大额资金使用等涉及全局的重大问题向社会公开发布和说明。

　　繁荣文化教育科技体育事业。实施大文化建设战略，创作文化精品，搞活文化市场，做大文化经济。开展社区、农村、校园、企业、家庭等多种文化活动，丰富城乡文化生活。调整中小学教育布局，抓好滨洲村九年一贯制等学校建设工程，全面完成农村学校D级危房改造，巩固提高“普九”成果，加强农村教育、职业教育和特殊教育。支持石油学院、八一农大搞好大学科技园、研究中心和实验基地建设，加大与哈工大等高校的合作力度，建好高新区创业服务中心，发展科技项目中试基地，加快产学研结合，研究开发一批项目，争取在石化、食品、医疗、新材料等领域取得实质性突破。建立公平统一的人才政策体系，研究引进外地人才、用好现有人才的具体办法，建立柔性流动的人才管理机制，充分发挥人才作用。启动建设奥林匹克花园，规划建设奥林匹克体育馆，办好市第九届地企联合运动会，开展群众体育活动，推动全民健身。

　　加强精神文明建设。深入开展全国文明城市创建，以弘扬大庆精神、铁人精神为主题，强化市民大庆意识、法律意识和公德意识教育，加强未成年人和大学生思想道德建设，切实提高市民素质。深入实施百事剖析、百项强市、百窗创优、百品兴市、百园建设、百面红旗、百区平安、百区改造、百件实事为内容的“九个百工程”。进一步巩固“双拥”模范城创建成果，抓好“双拥”共建。积极组织丰富多彩的群众性精神文明创建活动，全面推进文明家庭、文明社区、文明村镇、文明行业建设，逐步形成良好的社会风气，建设文明大庆。

　　加强社会治安综合治理。依法严厉打击各种刑事犯罪，坚持不懈地与“法轮功”等邪教组织作斗争，严密防范和坚决打击国内外敌对势力的破坏活动。认真抓好安全生产，落实安全生产责任制，强化安全生产措施，加强安全防火、交通安全、食品安全、药品安全工作，坚决防止重特大安全事故发生，营造安定的社会环境。

　　各位代表，石油石化大企业是大庆的根脉，地企关系是大庆最重要的生产关系，地企合作是大庆最重要的生产力。我们将一如既往地维护好、发展好地企关系，做到发展共促、压力共担、稳定共抓、城市共建，共同肩负起大庆可持续发展的重任。加深地企沟通。在经济发展、城市建设、维护稳定三个方面，完善高层协商和专项协作工作机制，对事关大庆区域统筹协调发展的大事要事，同谋划、共推进，增强发展整体合力。加大服务力度。主动到大企业走访调研，真心帮助大企业排忧解难。严厉打击盗抢原油和油田物资违法犯罪行为；细化征地补偿办法，帮助协调解决占用农田打井用地问题；配合油田公司进行占压油气管线、危害油田安全建筑的动迁改造；共同做好就业再就业和维护社会稳定工作，为大企业营造良好的生产经营环境。加强联合协作。共同向上争取资源型城市经济转型试点、天然气联合开发等政策，积极申报老工业基地振兴计划项目，继续搞好外围小油田联合开发。共同研究利用好大企业的物流和服务需求，开发建设一批配套加工、配套服务项目，实现大企业物资和服务供应本地化。共同研究衔接石化原料吃配的有效方法，争取在品种、数量、价格等方面向地方企业倾斜，大力发展石化产品精深加工产业。联手活化存量资产，支持大企业下属各生产经营单位重组改制，最大限度融合地企生产要素。继续加大协调合作力度，争取大企业在城市基础建设、绿化美化、泡泽治理、污染防治等方面的更多投入，共建美好家园。

　　各位代表，加速发展经济，努力改善民生，建设全面、协调、可持续发展的大庆，是全市人民面临的光荣而艰巨的任务，也是各级政府肩上的重大历史使命。我们要加强政府自身建设，树立正确的政绩观，始终坚持把发展作为第一要务，始终坚持执政为民，始终坚持调动一切积极因素，始终坚持努力维护大庆安定团结的大好局面，始终坚持依法行政、从严治政，努力创建学习型、服务型、创新型、法治型、廉洁型政府。解放思想，拓宽工作思路。做好政府工作关键在人，关键在人的思想观念、精神状态、工作作风和认知能力。要引导广大公务员努力克服计划经济的旧思维、旧模式、旧套路、旧方法，认真学习借鉴发达地区经验，创新思维，大胆开拓，用新思路、新机制、新办法破解前进中的难题，用改革的精神和勇气创造性开展工作。求真务实，提高行政效能。切实转变工作作风，深入基层群众，听民声、察民情、体民忧、解民难，多做事关大庆长远的事，多做人民群众期盼的事。建设政府办公自动化平台，推进网上审批；建设公共支付平台，逐步实现水、电、热、通信、有线电视等各类公共收费电子支付；建设公共信息服务平台，构建便民服务网络，建设“数字大庆”。开展好第一次全国经济普查工作，建立覆盖国民经济各个行业的数据库系统，健全统计监测和预报系统。加强财政预算管理和专项资金使用情况审计监督，提高财政资金使用效益。加强行政效能监察，开展专项监察和明查暗访，对政府部门工作作风、工作质量、工作效率全程监督，确保政令畅通，努力形成干事创业、务实高效的政务环境。从严治政，狠抓工作落实。强化工作落实责任制，对经济社会发展中的重点项目、大事实事采取专项推进办法，跟踪督办。敏于发现问题，敢于抓住问题，善于解决问题，在解决问题中抓好落实。教育引导广大公务员，务必保持谦虚谨慎、不骄不躁的作风，务必保持艰苦奋斗的作风，坚决反对追求表面政绩、急功近利的作风，少说多干，真抓实干。组织开展企业评价部门、群众评议干部活动，使干实事抓落实处于组织和群众的双重监督之下。不断提高公务员的创新能力、调查研究能力、沟通协调能力、公共服务能力和应对突发事件能力。廉洁勤政，维护政府形象。认真落实廉政建设责任制，坚决按照“四承诺”、“三树立”、“两禁止”要求办事。深入开展警示教育，防微杜渐，警钟长鸣，增强公务员的自我约束能力和拒腐防变能力，始终保持昂扬向上的精神状态，始终保持人民公仆本色。

　　各位代表，我市正处在加快发展的关键时期，任务十分艰巨。让我们在市委的领导下，紧紧依靠全市人民，发扬大庆精神、铁人精神，解放思想，精诚团结，与时俱进，开拓创新，努力构建大庆全面发展战略新高地，共同创造我们的幸福生活和美好未来！
